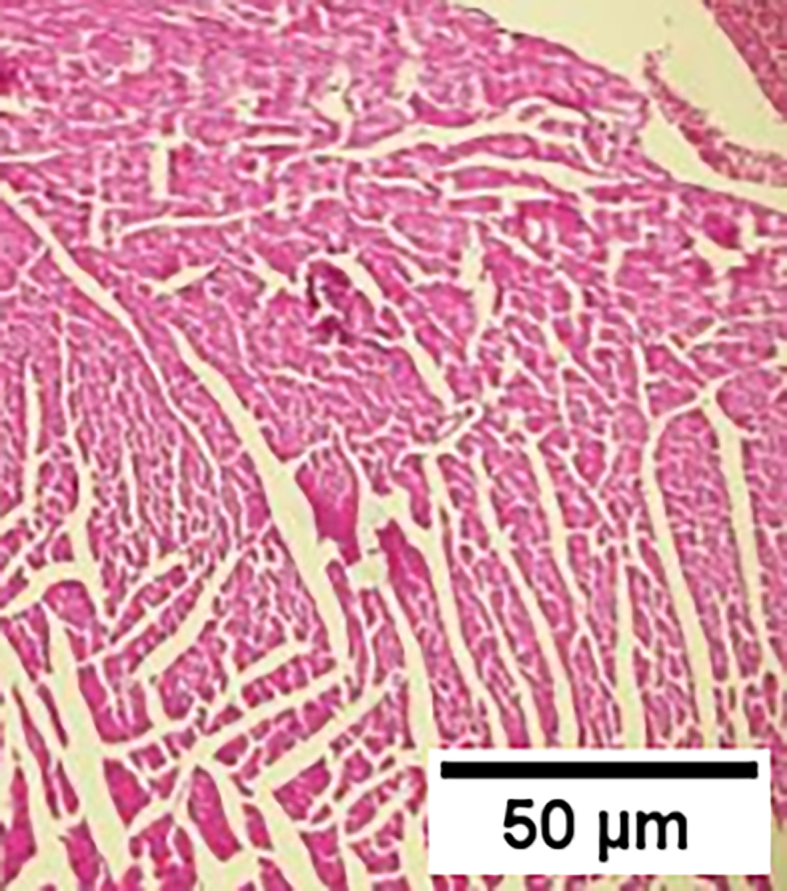# Dose-dependent antidiabetic and multiorgan protective effects of *Ziziphus abyssinica* methanolic extract in streptozotocin-induced diabetic mice

**DOI:** 10.1016/j.jaim.2026.101318

**Published:** 2026-05-20

**Authors:** Tsegay Beyene Weldemariam, Abebaye Aragaw Leminie, Wossene Habtu Tadesse, Worku Gemechu Lemmi, Samuel Woldekidan Hirpesa, Rekik Ashebir Muluye, Abiy Abebe gelagle, Sofia Yimam Hussen, Getahun Tsegaye Dibaba, Beza Tasew Degefu, Tesfaye Tolessa Dugul

**Affiliations:** a School of Medicine, College of Health Sciences, Aksum University, Ethiopia; b School of Medicine, College of Health Sciences, Addis Ababa University, Ethiopia; c National Clinical Chemistry Reference Laboratories, Ethiopia Public Health Institute, Ethiopia; d Traditional and Modern Medicine Directorates, Armauer Hansen Research Institute, Ethiopia; e Non-communicable Division, Armauer Hansen Research Institute, Ethiopia

**Keywords:** *Ziziphus abyssinica*, Antidiabetic, Streptozotocin (STZ), Hyperglycemia

## Abstract

**Background:**

Diabetes mellitus (DM) is a global health challenge, with rising prevalence and significant economic burden, particularly in low- and middle-income countries. Current synthetic antidiabetic drugs, though effective, are associated with adverse effects, prompting interest in plant-based alternatives.

**Objectives:**

This study evaluated the antidiabetic potential of *Ziziphus abyssinica* methanolic extract (ZAE) in streptozotocin (STZ)-induced diabetic mice.

**Methods:**

Male Swiss Albino mice were grouped into normal control, diabetic control, glibenclamide (5 mg/kg), and ZAE-treated (200, 400, 600 mg/kg) groups. Parameters were monitored for 28 days.

**Results:**

ZAE at 600 mg/kg normalized blood glucose (148.7 ± 11.0 mg/dL vs. normal control group 148.0 ± 3.0 mg/dL, ∗*P*∗ >0.05), achieving a 66 % reduction (∗*P* ∗ < 0.001) versus diabetic controls (437.2 ± 43.6 mg/dL). Body weight recovered fully (40.5 ± 1.9 g vs. 41.2 ± 1.2 g, ∗*P* ∗ >0.05), contrasting with a 25 % loss in diabetic controls (∗*P*∗ < 0.001).

Lipid profiles improved, with HDL rising (61.8 ± 3.9 vs. 33 ± 2.4 mg/dL, ∗*P*∗ = 0.002) and LDL declining (37.5 ± 2.4 vs. 58.8 ± 3.7 mg/dL, ∗*P*∗ = 0.002). Liver (ALT, AST, ALP) and renal (creatinine, urea) markers normalized (∗*P*∗ < 0.05).

Histopathology revealed β-cell preservation (∗*P*∗ < 0.01) and, specifically at the 600 mg/kg, a marked reduction in hepatic inflammation and renal protection.

**Conclusion:**

The 600 mg/kg ZAE demonstrated novel, dose-dependent efficacy not only in glycemic control but also in pancreatic β-cell preservation and multiorgan protection, showing significant benefits in renal and cardiac tissues where glibenclamide was ineffective. These findings suggest the traditional use of *Ziziphus abyssinica* and highlight the potential of 600 mg/kg regimen to provide concurrent glycemic control and multi-organ protection.

## 1 Introduction

Diabetes mellitus (DM) is a chronic metabolic disorder characterized by hyperglycemia due to insulin deficiency, insulin resistance, or both. It affects over 537 million adults globally, with projections suggesting a rise to 783 million by 2045 [1], posing a significant public health and economic burden [2]. The economic impact is particularly severe in low- and middle-income countries, where diabetes management costs can consume up to 15 % of healthcare budgets [2]. Type 2 diabetes (T2DM), accounting for 90 % of cases, is driven by lifestyle factors and genetic predisposition, while Type 1 diabetes (T1DM) results from autoimmune destruction of pancreatic β-cells [3]. Recent genome-wide association studies have identified over 400 genetic loci associated with T2DM risk, highlighting the complex interplay between genetic and environmental factors [4]. Chronic hyperglycemia leads to severe complications, including nephropathy, neuropathy, and cardiovascular diseases, underscoring the need for effective therapeutic strategies [5].

Current diabetes management relies on synthetic drugs like metformin and glibenclamide, which improve insulin sensitivity or secretion but are associated with adverse effects such as hypoglycemia, weight gain, and gastrointestinal disturbances [6]. These limitations have spurred interest in plant-derived antidiabetic agents, which often exhibit multimodal mechanisms of action and fewer side effects [7]. The World Health Organization estimates that approximately 80 % of the global population relies on traditional plant-based medicines for primary healthcare [8,9]. Ethnobotanical studies highlight the use of medicinal plants like *Ziziphus abyssinica* in traditional African medicine for managing diabetes, attributed to their rich phytochemical profiles [10].

Recent phytochemical analyses of *Ziziphus species* have identified over 50 bioactive compounds with potential antidiabetic properties, including flavonoids, alkaloids, and triterpenes [11,12].

Streptozotocin (STZ)-induced diabetic models are widely employed to study diabetes pathophysiology and therapeutic interventions. STZ selectively destroys pancreatic β-cells via DNA alkylation and oxidative stress, mimicking T1DM, while repeated low-dose STZ protocols can induce insulin resistance similar to T2DM [13]. Recent modifications to the STZ model, including combination with high-fat diets, have improved its relevance for studying T2DM pathogenesis and complications [14]. This model is particularly valuable for evaluating plant extracts, as it replicates key features of human diabetes, including hyperglycemia, weight loss, and organ damage [15]. The STZ model's reliability has been demonstrated in studies assessing β-cell regeneration and hepatorenal protection by natural compounds [16].

*Ziziphus,* a member of the Rhamnaceae family, is traditionally used across Africa for its antidiabetic, anti-inflammatory, and antioxidant properties [11]. The genus Ziziphus includes over 100 species, with at least 20 documented in traditional medicine for metabolic disorders [11]. Phytochemical analyses reveal the presence of flavonoids, alkaloids, and phenolic acids, which are known to enhance insulin secretion, reduce hepatic glucose output, and mitigate oxidative stress [17]. Recent studies have shown that these compounds can modulate key signaling pathways involved in glucose homeostasis, including AMPK and PPAR-γ activation [17]. Methanolic extracts of *Z. abyssinica* have shown promising hypoglycemic effects in preliminary studies, likely due to their ability to scavenge free radicals and modulate glucose metabolism pathways [18]. A comprehensive understanding of its pharmacokinetics and tissue distribution would be valuable for optimizing therapeutic applications [19].

However, systematic and comprehensive investigations into its dose-dependent efficacy and concurrent organ-specific protective effects remain limited. This study therefore aimed to perform an integrated pathological evaluation of the antidiabetic potential of *Ziziphus abyssinica* methanolic extract (ZAE) in STZ-induced diabetic mouse model, focusing on glycemic control, body weight dynamics, and histopathological outcomes. By comparing 200 mg/kg, 400 mg/kg, and 600 mg/kg doses against glibenclamide (positive control), we sought to elucidate its dose-response relationship and therapeutic mechanisms. The findings could validate its traditional use and provide a scientific basis for developing plant-based antidiabetic therapies [20]. Future studies should explore the potential synergistic effects of ZAE with conventional antidiabetic drugs, as combination therapies often show enhanced efficacy with reduced side effects [7].

## 2 Materials and methods

### 2.1 Materials

#### 2.1.1 Chemicals and reagents

1. For Plant Material Extraction:

Methanol (Absolute, ≥99.8 % purity, analytical grade) for preparing the 70 % v/v extraction solvent.

Distilled Water (Prepared in-house using a Millipore water purification system or equivalent).

2. For Induction of Diabetes (STZ Model):

Streptozotocin (STZ), highly labile, requires cold chain storage.

Sodium Citrate Dihydrate (for preparing 0.1 M citrate buffer).

Citric Acid (Anhydrous) (for preparing 0.1 M citrate buffer and adjusting pH to 4.5).

Sucrose (for preparing 10 % sucrose solution to counter acute hypoglycemia post-STZ injection).

3. For Treatment Administration:

Glibenclamide, USP standard, for the positive control group (5 mg/kg).

Vehicle: Distilled Water (for dissolving glibenclamide and as the vehicle for the extract and control groups).

4. For Blood Collection:

EDTA (K2EDTA or K3EDTA) Coated Tubes (lavender top) for whole blood collection for CBC analysis.

Serum Separator Tubes (SST) for blood chemistry analysis.

5. For Euthanasia:

Compressed Carbon Dioxide (CO_2_) Gas (Medical grade or research grade) in a regulated tank with a controlled flow valve.

### 2.2 Plant collection and identification

Leaf and root part of the *Ziziphus abyssinica* medicinal plant was collected from rural areas of Axum in the Tigray region of Ethiopia (14°07′15″N, 38°43′40″E; elevation: 2131 m) during the dry season (September–October 2024) to minimize variability in phytochemical content linked to seasonal precipitation. The plant samples were identified by comparison with herbarium samples housed in the Department of Botany at Addis Ababa University. The scientific name “*Ziziphus abyssinica* Hochst. ex A. Rich”. was confirmed, and its voucher sample (TB001) was authenticated by Melaku Wendafrash, who is a botanist at the Addis Ababa University Herbarium Center. The leaf part of the plant was selected based on the basis of its traditional ethnopharmacological uses, which include analgesic effects; management of diabetes nephropathy; diabetes mellitus; diabetic wound healing [21].

### 2.3 Methanolic leaf extraction

The leaves of *Z. abyssinica* were washed thoroughly to remove debris, shade-dried at room temperature 25–30 °C for 10 days, and ground into a fine powder using an electric grinder, followed by sieving 60-mesh sieve, 0.25 mm pore size [22] For methanolic extraction, 100 g of powdered leaves were macerated in 1200 mL of 70 % methanol (1:12, w/v) for 48 h at room temperature with occasional stirring, based on preliminary optimization for maximizing polar/nonpolar phytochemical recovery. The mixture was then shaken for 2 h using an orbital shaker 150 rpm, filtered through Whatman No. 1 filter paper, and concentrated under reduced pressure 40 °C, 60 rpm using a rotary evaporator [23]. The extraction yield was calculated as 31.46 ± 0.16 % w/w.

Methanol (70 %) was selected for its high efficiency in extracting bioactive compounds with established antidiabetic properties, such as flavonoids, alkaloids, and phenolic acids [24]. The resulting extract was stored at −20 °C in airtight amber vials to prevent degradation for further antidiabetic assays [25]. It is important to note that while the employed evaporation process is designed to remove solvent, the final extract was not specifically analyzed for residual methanol content.

2.4

#### 2.4.1 Euthanasia and sample collection

Upon completion of the experimental period, all animals were humanely euthanized via carbon dioxide (CO_2_) inhalation, following approval from the Addis Ababa University Ethical Review Board. The procedure adhered to AVMA Guidelines (2020 Edition) [26] and international standards, employing a controlled gradual fill rate (30–70 % chamber volume/min) to ensure stress-free unconsciousness prior to potential nociception (>40 % CO_2_). This method was selected for its rapid anesthetic effect, cost-efficiency, and minimal impact on study parameters. To further reduce stress, animals remained in their home cages during the procedure, with continuous chamber monitoring to guarantee uniform gas distribution. Death was confirmed by absence of cardiac activity and respiratory arrest [27]**.**

Immediately post-euthanasia, cardiac puncture was performed by trained personnel to collect high-volume, uncontaminated blood samples avoiding stress-related biochemical artifacts. Critical tissues (liver, kidney, heart and pancreas) were rapidly excised to prevent autolysis and preserve morphological integrity for histopathological analysis [28].

All procedures complied with: OECD Guidelines for Chemical Testing and Institutional Ethical Guidelines (Addis Ababa University; Protocol #018/24/physio).

### 2.5 Animal study

#### 2.5.1 Streptozotocin-induced diabetic model in Swiss Albino mice

**Fig. 1:**
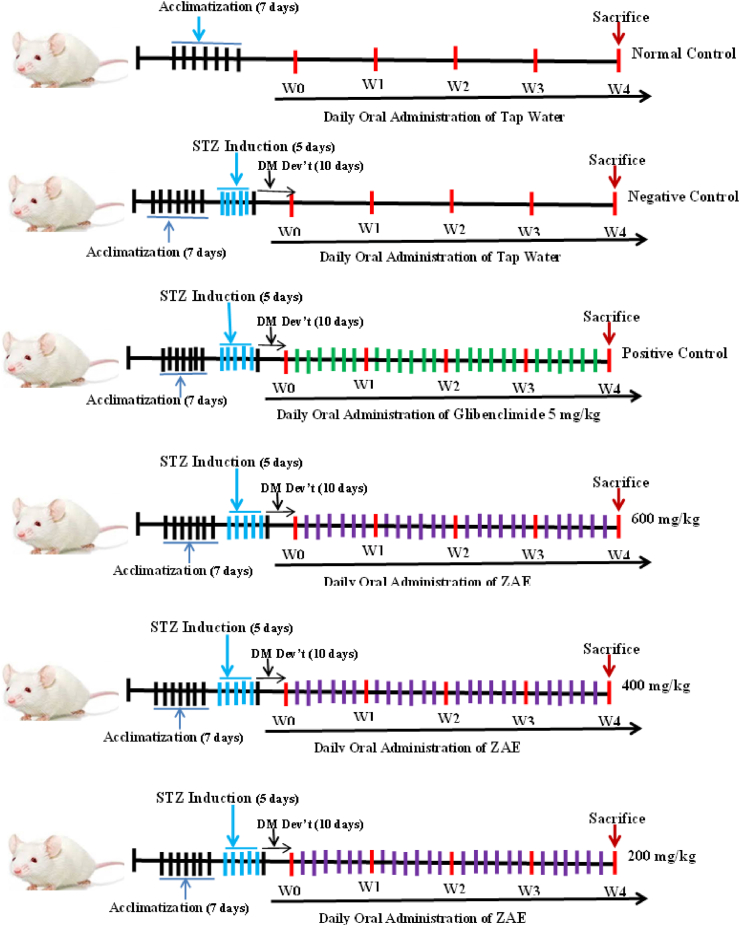
Experimental timeline of STZ-Induced diabetes model and treatment protocol

Ten-week-old male Swiss Albino mice (∼25 g) were housed in groups of six per cage under controlled conditions (24 ± 1 °C, 55 ± 5 % humidity, 12-hr light-dark cycle) with ad libitum access to standard chow and water for a 7-day acclimatization period [29]. Four hours prior to STZ administration, food was withdrawn & water remained available, and mice were weighed and stratified into control and experimental groups to ensure uniform weight distribution [30]. A freshly prepared 0.1 M sodium citrate buffer (pH 4.5) was used to dissolve STZ (20 mg/mL), with the buffer prepared by mixing sodium citrate dihydrate (2.941 g/100 mL) and citric acid (1.921 g/100 mL), adjusted to pH 4.5 by combining 30 mL sodium citrate with 25 mL citric acid. STZ solutions were prepared under dim light and kept on ice to minimize degradation [31]. The STZ solution was administered intraperitoneally (i.p.) at 40 mg/kg (1.0 mL/100 g body weight) for five consecutive days, while normal control mice received an equivalent volume of citrate buffer. Immediately post-injection, mice were provided normal chow and 10 % sucrose water to counteract acute hypoglycemia and monitored every 2 h for 12 h for signs of hypo activities [32]. On Day 3, sucrose water was replaced with regular water [14]. To confirm diabetes induction, mice were fasted for 6 h on Day 10 post-STZ, and blood glucose was measured via tail-vein sampling using a OneTouch Verio® glucometer; hyperglycemia was defined as >200 mg/dL, with severe diabetes (blood glucose 300–600 mg/dL) typically evident by Week 3. Mice with blood glucose <200 mg/dL post-induction were excluded from the study (see Fig. 1) [33].

#### 2.5.2 Treatment and control groups

The study comprised six groups with a sample size of n = 6 mice per group. The sample size was determined based on established standards for preclinical diabetes research in murine models, which consistently demonstrate that n = 6 provides sufficient statistical power to detect significant changes in key metabolic parameters w hile adhering to the ethical principles of the 3Rs (Replacement, Reduction, and Refinement) [3,30,34]. All procedures were planned and reported in accordance with the ARRIVE 2.0 guidelines [35]**.** The groups included three experimental and three control groups. Experimental groups received daily oral gavage of *Z. abyssinica* extract (ZAE) at 200, 400, and 600 mg/kg doses. Control groups included: a non-diabetic normal control (receiving distilled water), a positive control (STZ-induced diabetic mice treated with glibenclamide at 5 mg/kg/day, orally), and a diabetic negative control (STZ-induced mice receiving distilled water). Treatments were administered daily for 28 days, with body weight and fasting blood glucose monitored weekly (days 0, 7, 14, 21, and 28) to assess efficacy and safety [3,25,30].

#### 2.5.3 Body weight measurements

Body weight measurements were conducted weekly for five weeks in six experimental groups (n = 6 Swiss albino mice/group) using a calibrated analytical balance. Mice were weighed at a consistent time to minimize circadian variability, with care taken to avoid stress during handling [36,37]. Data were analyzed using repeated-measures ANOVA after confirming sphericity (Mauchly's test, followed by Tukey's post-hoc tests for group comparisons at each timepoint (α = 0.05). All procedures adhered to Animal Ethics Committee guidelines.

#### 2.5.4 Blood glucose monitoring

Fasting blood glucose levels were measured weekly for five weeks via tail vein sampling using an FDA-approved glucometer OneTouch Verio® glucometer between 010:00–11:00 to minimize circadian variability [38]. Mice were fasted for 6 h prior to measurement with free access to water. Six experimental groups (n = 6/group) were analyzed [39]. Data were analyzed using repeated-measures ANOVA in SPSS version 26 after confirming sphericity. The model assessed within-subjects (time) and between-subjects (group) effects, plus their interaction. Post-hoc Tukey's HSD tests (α = 0.05, two-tailed) compared groups at each time point, with effect sizes reported as partial η^2^. Quadratic contrast analysis evaluated dose-response trends.

#### 2.5.5 Hematological analysis

At terminal sacrifice (Day 28), whole blood was collected *using a 25G needle* via cardiac puncture into EDTA-coated tubes, and analyzed within 2 h using DxH 800 Hematology Analyzer (Beckman Coulter, USA calibrated daily with manufacturer-provided controls [40]. Complete blood counts quantified: erythrocyte parameters (RBC, hemoglobin [HGB], hematocrit [HCT], MCV, MCH, MCHC, RDW), leukocyte subsets (WBC, neutrophils, lymphocytes [LYM], monocytes, eosinophils, basophils), and platelets (PLT) [41]. Six experimental groups (n = 6/group) were assessed. Non-normally distributed data (Shapiro-Wilk test, *P* < 0.05) were analyzed using Kruskal-Wallis with Bonferroni-adjusted Dunn's post-hoc tests (α = 0.05), while parametric comparisons employed one-way ANOVA (Tukey's correction). Effect sizes were reported as η^2^ for parametric and ε^2^ for non-parametric tests. All measurements followed ICSH guidelines, with outlier exclusion criteria (>3 SD from group mean).

#### 2.5.6 Blood chemistry analysis

Blood samples were collected via cardiac puncture at terminal sacrifice (Day 28) following a 6-h fast, allowed to clot at room temperature for 30 min, and centrifuged at 3000×*g* for 15 min to isolate serum. Biochemical parameters were quantified using standardized automated assays (Cobas c501 analyzer, Roche Diagnostics): glucose, ALT/AST, ALP, bilirubin, lipids, and electrolytes. All assays included daily calibration with manufacturer-provided standards and internal quality controls (normal/pathological ranges). Liver function (ALT, AST, ALP), glucose metabolism, lipid profile (total cholesterol, HDL, LDL, triglycerides), renal markers (creatinine, urea), and electrolytes (Na^+^, K^+^, Cl^−^) were analyzed in six experimental groups (n = 6/group) [41]. Non-normally distributed data (Shapiro-Wilk test, *P* < 0.05) were analyzed using Kruskal-Wallis with Dunn's post-hoc tests (α = 0.05, two-tailed), while parametric comparisons employed one-way ANOVA with Tukey's correction. Inter-group differences were considered significant at *P* < 0.05.

#### 2.5.7 Histopathological examination

Pancreatic, hepatic, renal, and cardiac tissues were harvested immediately post-euthanasia, fixed in 10 % neutral-buffered formalin for 48 h, and processed through graded ethanol series for paraffin embedding. Sections (4 μm thickness) were stained with hematoxylin and eosin (H&E) using standard protocols (hematoxylin: 5 min; eosin: 3 min) [42] and examined under bright-field microscopy (Nikon Eclipse E200, 40–400 × magnification). Pancreatic islet morphology was evaluated for: (1) islet number/area, (2) β-cell density, relative to islet area, and (3) lymphocytic infiltration, insulitis. Liver sections were assessed for architectural distortion (necrosis, steatosis), inflammatory foci (periportal lymphocytes), and cellular stress markers (pyknosis, hyperchromasia). Kidney analysis focused on glomerular integrity, tubular vacuolization, and perivascular inflammation, while cardiac sections were screened for myocyte disarray, vascular abnormalities, and foam cell infiltration. All histopathological evaluations were performed by two blinded investigators. Representative photomicrographs were captured using a digital camera under standardized lighting conditions.

#### 2.5.8 Organ collection and weight analysis

Pancreas, liver, kidneys, and heart were promptly excised, carefully rinsed in ice-cold phosphate-buffered saline (PBS, pH 7.4) to remove residual blood, and gently blotted dry on sterile filter paper to eliminate excess moisture [43]. Each organ was weighed immediately using a precision analytical balance; sensitivity ±0.1 mg, with absolute weights recorded in milligrams (mg) to minimize post-excision variability. To ensure consistency, all weighings were performed by the same investigator under identical conditions (room temperature: 22 ± 1 °C; humidity: 50 ± 5 %) [44]. Organ weight data were presented as mean ± standard deviation (SD) with individual ranges, using N = 6 biologically independent samples per group. Statistical comparisons were performed using one-way ANOVA followed by Tukey's post-hoc test to assess inter-group differences, with significance thresholds set at ∗ *P* < 0.05, ∗∗*P* < 0.01, and ∗∗∗*P* < 0.001 relative to the Normal Control group. This stringent analytical approach ensured robust quantification of STZ-induced pathological changes such as pancreatic atrophy, hepatomegaly, and treatment-associated recoveries, as evidenced by the dose-dependent normalization trends in 600 mg/kg and positive control groups [45].

## 3 Results

### 3.1 Body weight

Body weight was measured over five weeks in six groups (n = 6/group): Diabetic control, Normal control, Positive control (Glibenclamide, 5 mg/kg), ZAE 200 mg/kg, ZAE 400 mg/kg, and ZAE 600 mg/kg. One animal in the diabetic control group succumbed to the disease prior to the end of the study period; data from this animal were included in the analysis where post-mortem samples allowed. The data are presented in Table 1.

**Table 1:** Mean body weight (g) ± SD by group and week.

Group	Baseline (W0)	Week 1 (W1)	Week 2 (W2)	Week 3 (W 3)	Week 4 (W 4)
Normal Control	35.7 ± 2.1	36.7 ± 1.8	36.5 ± 1.9	42.2 ± 1.7	41.2 ± 1.2
High Dose	36.0 ± 1.4	35.3 ± 2.2	35.0 ± 1.8	40.5 ± 1.9	40.5 ± 1.9
Middle Dose	35.0 ± 2.4	33.7 ± 1.5	33.8 ± 1.5	34.5 ± 1.9	34.8 ± 2.1
Low Dose	35.5 ± 1.9	33.5 ± 1.9	30.5 ± 1.9	32.7 ± 1.6	32.8 ± 3.6
Positive Control	34.0 ± 1.4	36.0 ± 1.4	36.5 ± 1.9	36.5 ± 1.9	38.5 ± 1.9
Diabetic Control	36.7 ± 2.7	30.2 ± 1.5	30.7 ± 2.2	30.2 ± 2.3	27.5 ± 1.9

**Notes**: **Bold**: Key comparisons (High Dose ≈ Normal Control at W4). Data are mean ± SD; Positive Control received glibenclamide (5 mg/kg). W0: Baseline.

### 3.2 Blood glucose

Blood glucose levels were measured from week 0 to week 4. The longitudinal profile for all groups is shown in Table 2.

**Table 2:** Blood glucose levels (mg/dL) by group and experimental week (mean ± SD).

Group	Week 0	Week 1	Week 2	Week 3	Week 4
Low Dose	226.3 ± 16.9	210.7 ± 7.0	203.0 ± 8.7	195.7 ± 6.5	200.3 ± 9.5
Middle Dose	238.8 ± 11.9	205.8 ± 4.4	192.7 ± 5.9	187.2 ± 12.0	183.7 ± 15.1
High Dose	245.0 ± 9.9	198.8 ± 5.2	169.3 ± 16.3	152.7 ± 45.3	148.7 ± 11.0
Diabetic Control	238.5 ± 12.2	255.2 ± 3.6	353.7 ± 44.2	423.3 ± 36.3	437.2 ± 43.6
Positive Control	240.8 ± 3.3	197.3 ± 11.9	188.8 ± 8.3	166.7 ± 17.5	180.8 ± 12.0
Normal Control	137.8 ± 12.3	148.2 ± 17.4	135.8 ± 9.6	150.7 ± 20.9	148.0 ± 3.0

**Notes**: **Bold** highlights key comparisons (High Dose vs. Diabetic Control at Week 4). *Positive Control*: Glibenclamide (5 mg/kg). Data are mean ± SD; ∗n∗ = 6/group.

### 3.3 Biochemical parameters

Serum biochemical parameters were analyzed at terminal sacrifice (day 28). The data are presented in Table 3.

**Table 3:** Biochemical Parameters in Treatment and experimental Mice at 28th day (Mean ± SD).

Parameters	LD	MD	HD	DC	PC	NC
Glucose (mg/dL)	131.3 ± 5.9	127.3 ± 1.6	120.5 ± 3.9	210.3 ± 9.2	152.7 ± 6.3	117.2 ± 1.8
ALT (IU/L)	53.5 ± 2.3	51.3 ± 2.3	48.5 ± 2.7	61.8 ± 4.8	46.5 ± 4.7	46 ± 2.8
AST (IU/L)	160.2 ± 1.7	159.5 ± 1.9	136.2 ± 28.8	166.5 ± 3.1	113.7 ± 24.7	99.8 ± 13.2
ALP (IU/L)	194.7 ± 9	195.3 ± 7.2	146.7 ± 6.3	226.3 ± 4.1	142.2 ± 5	140.7 ± 4
D.BIL (mg/dL)	0.4 ± 0.1	0.3 ± 0.1	0.2 ± 0.1	0.6 ± 0.1	0.2 ± 0.1	0.1 ± 0.1
I.BIL (mg/dL)	0.6 ± 0.1	0.5 ± 0.1	0.4 ± 0.1	0.7 ± 0.1	0.5 ± 0.1	0.2 ± 0.1
Albumin (g/dL)	3.0 ± 0.1	2.7 ± 0.2	2.6 ± 0.1	3.1 ± 0.1	2.7 ± 0.1	2.6 ± 0.1
Total protein(g/dL)	5.2 ± 0.1	5.2 ± 0.1	5.2 ± 0.1	5.3 ± 0.2	5.1 ± 0.1	5.2 ± 0.1
Urea (mg/dL)	62.0 ± 0.9	58.2 ± 1.7	55 ± 2.1	54.8 ± 3.1	56.7 ± 4	54.5 ± 3.3
Creatinine (mg/dL)	0.4 ± 0.1	0.5 ± 0.1	0.3 ± 0.1	0.5 ± 0.1	0.5 ± 0.1	0.4 ± 0.1
Triglyceride(mg/dL)	160.5 ± 2.4	153.8 ± 7.3	143.3 ± 4.6	167.5 ± 5.2	160.2 ± 1.2	129.8 ± 4
Cholesterol(mg/dL)	96.3 ± 1.6	93.0 ± 1.4	82.5 ± 3.5	99.8 ± 1.9	94.2 ± 1.5	79.2 ± 2.5
LDL (mg/dL)	43.0 ± 1.7	40.3 ± 1.6	37.5 ± 2.4	58.8 ± 3.7	41.7 ± 1.8	33.8 ± 1.9
HDL (mg/dL)	40.8 ± 1.5	45.7 ± 3.6	61.8 ± 3.9	33 ± 2.4	50.5 ± 5.8	93 ± 2.4
LDH (IU/L)	444.0 ± 191.5	447.2 ± 43.2	290.3 ± 71.7	777 ± 63.7	416.5 ± 20.4	237.5 ± 26.5
Sodium (mEq/L)	147.2 ± 5.4	141.5 ± 3.7	132.2 ± 3.4	141.2 ± 11.2	137 ± 9.3	132.3 ± 3.8
Potassium (mEq/L)	2.8 ± 0.1	2.62 ± 1.8	3.1 ± 0.2	3.5 ± 0.4	3.1 ± 0.6	3.2 ± 0.2
Chloride (mEq/L)	91.5 ± 5.2	101.3 ± 2.6	120.5 ± 6.4	58.5 ± 6.9	90.7 ± 8	118.5 ± 6

Abbreviations.

LD:Low Dose; Mice Treated with *Ziziphus abyssinica* Extract (200 mg/kg).

MD:Middle Dose; Mice Treated with *Ziziphus abyssinica* Extract (400 mg/kg).

HD:High Dose; Mice Treated with *Ziziphus abyssinica* Extract (600 mg/kg).

DC:Diabetic Control; Mice Treated with vehicle (distilled water).

PC:Positive Control; Mice Treated with glibenclamide (5 mg/kg).

NC:Normal Control; Mice Treated with vehicle (distilled water).

ALT (alanine aminotransferase), AST (aspartate aminotransferase), ALP (alkaline phosphatase), LDH (lactate dehydrogenase), LDL (low-density lipoprotein), HDL (high-density lipoprotein), D.BIL (Direct Bilirubin), I.BIL **(**Indirect Bilirubin) (***N*** = 6 mice per group.

**Glucose:** The ZAE 600 mg/kg group had a blood glucose level of 120.5 ± 3.9 mg/dL, compared to 210.3 ± 9.2 mg/dL in the diabetic control group and 152.7 ± 6.3 mg/dL in the positive control (Glibenclamide) group.

**Liver Function:** Alanine aminotransferase (ALT) levels were 53.5 ± 2.3 IU/L, 50.2 ± 3.1 IU/L, and 48.5 ± 2.7 IU/L in the ZAE 200, 400, and 600 mg/kg groups, respectively, compared to 61.8 ± 4.8 IU/L in the diabetic control group. Aspartate aminotransferase (AST) levels were 136.2 ± 28.8 IU/L in the ZAE 600 mg/kg group. Lactate dehydrogenase (LDH) levels were 444.0 ± 191.5 IU/L, 350.8 ± 92.4 IU/L, and 290.3 ± 71.7 IU/L in the ZAE 200, 400, and 600 mg/kg groups, respectively.

**Lipid Profile:** Total cholesterol levels were 96.3 ± 1.6 mg/dL, 89.5 ± 2.8 mg/dL, and 82.5 ± 3.5 mg/dL in the ZAE 200, 400, and 600 mg/kg groups, respectively. High-density lipoprotein (HDL) levels were 40.8 ± 1.5 mg/dL, 51.3 ± 2.2 mg/dL, and 61.8 ± 3.9 mg/dL in the ZAE 200, 400, and 600 mg/kg groups, respectively, compared to 50.5 ± 5.8 mg/dL in the positive control group.

**Renal Function & Electrolytes:** Creatinine (0.3–0.5 mg/dL) and urea (55–62 mg/dL) levels across all ZAE-treated groups were within the range of the normal control group. Serum potassium was 2.62 ± 1.8 mEq/L in the ZAE 400 mg/kg group.

**Bilirubin:** Total bilirubin levels were 0.6 ± 0.1 mg/dL, 0.5 ± 0.1 mg/dL, and 0.4 ± 0.1 mg/dL in the ZAE 200, 400, and 600 mg/kg groups, respectively.

Hematological Parameters.

**Fig. 2:**
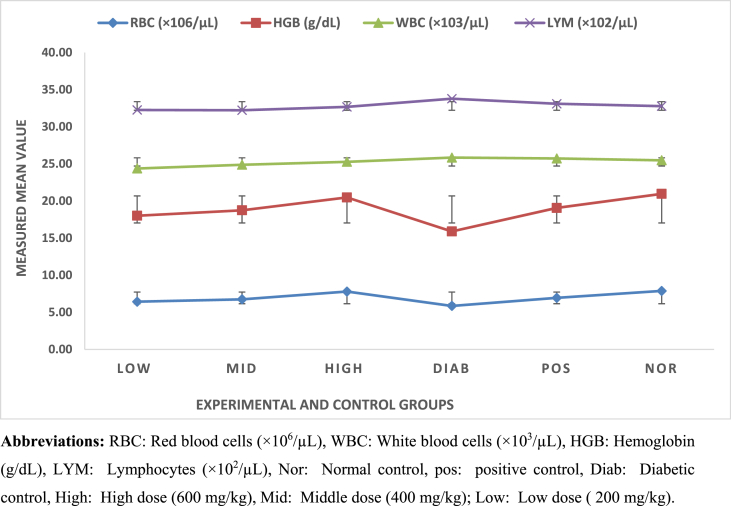
Hematological parameters in Swiss albino mice across experimental and control groups. Lines represent mean ± SEM. (For interpretation of the references to colour in this figure legend, the reader is referred to the Web version of this article.) Abbreviations: RBC: Red blood cells ( × 10^6^/μL), WBC: White blood cells ( × 10^3^/μL), HGB: Hemoglobin (g/dL), LYM: Lymphocytes ( × 10^2^/μL), Nor: Normal control, pos: positive control, Diab: Diabetic control, High: High dose (600 mg/kg), Mid: Middle dose (400 mg/kg); Low: Low dose (200 mg/kg).

A complete blood count was performed on day 28. The data for lymphocytes (LYM), white blood cells (WBC), hemoglobin (HGB), and red blood cells (RBC) are presented in Fig. 2.

### 3.4 Histopathological analysis

1) Pancreas Morphology

Histological analysis of pancreatic tissue revealed significant structural alterations induced by streptozotocin (STZ) treatment, which were mitigated by ZAE and glibenclamide treatment.

**Fig. 3:**
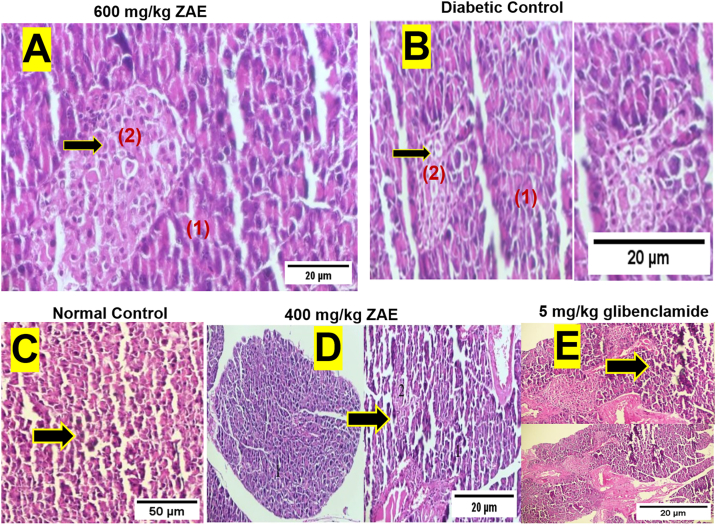
**Protective effects of ZAE on pancreatic islet morphology in STZ-induced diabetic mice.** Representative H&E-stained pancreatic sections (40× magnification) show the condition of islets across treatment groups. **(A)** Mice treated with 600 mg/kg ZAE exhibit a pancreatic islet with preserved architecture and normal cellularity, indicating a potent protective effect (2), alongside normal exocrine acini (1). **(B)** In contrast, the Diabetic Control group displays a severely damaged islet, exhibiting hallmark pathological features including significant shrinkage (atrophy) (2), loss of beta-cells (hypocellularity), and lymphocytic infiltration (insulitis). **(C)** The Normal Control shows an islet with typical size and structure. **(D)** A lower 400 mg/kg ZAE dose resulted in an islet with milder damage, suggesting a partial protective effect. **(E)** The standard drug glibenclamide (5 mg/kg) also preserved islet architecture, similar to the high-dose ZAE. **Arrows** point to representative islets. Scale bar, 20 μm.

Pancreatic islets in untreated STZ-induced diabetic control mice exhibited severe pathological changes compared to the non-diabetic healthy controls**.** These changes included a significant reduction in islet number and volume, decreased cellularity within the remaining islets, and prominent lymphocytic infiltration (insulitis) (Fig. 3-B).

Treatment with ZAE showed a dose-dependent protective effect on pancreatic islets. Mice treated with 400 mg/kg ZAE still showed significant structural alterations in the islets of Langerhans, including a reduction in number and volume, decreased size, and lymphocytic infiltration (Fig. 3-D). In contrast, pancreatic tissue from mice treated with the 600 mg/kg dose of ZAE showed markedly preserved architecture. The number and volume of islets were comparable to those of non-diabetic controls, and the cellular population within the islets was largely maintained (Fig. 3-A).

This preservation effect was also observed in the positive control group. The architecture of the islets in STZ-induced diabetic mice treated with 5 mg/kg glibenclamide was more preserved than in the untreated diabetic controls, exhibiting larger islet volume, greater cellularity, and reduced lymphocytic infiltration (Fig. 3-E). As expected, pancreatic tissue from non-diabetic healthy control mice displayed typical architecture of the exocrine acini and well-defined, numerous islets of Langerhans with high cellular density (Fig. 3-C).

2. Liver Morphology

Histopathological analysis revealed a complex, non-linear dose-response in the liver. While 200 and 400 mg/kg of ZAE were associated with signs of hepatic injury, 600 mg/kg unexpectedly demonstrated a significant protective effect. In the dose group 200 mg/kg, hepatic injury was evident as a multifocal pattern of lymphocytic infiltration localized to portal and periportal regions. This was accompanied by architectural distortion, necrotic hepatocyte foci, dilated sinusoids with microvesicular steatosis, and cellular stress markers including hyperchromatic nuclei and pyknosis.

The dose 400 mg/kg resulted in a marked intensification of lesions. The inflammatory response, characterized by dense perivascular lymphocytic infiltration, was more severe and widespread. Architectural distortion increased, with larger and more numerous necrotic foci. The emergence of perivenular inflammation indicated an expansion of the injury pattern towards the central vein, confirming a dose-dependent exacerbation of toxicity.

**Fig. 4:**
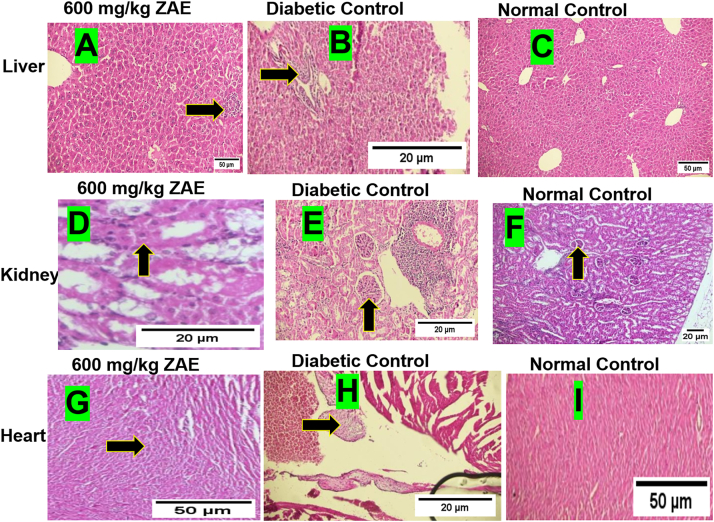
Histopathological **assessment of liver, kidney, and cardiac tissues following experimental treatment. (A**–**C)** Representative H&E-stained liver sections. **(A)** 600 mg/kg treatment (10x) showing preserved hepatic architecture with only mild perivascular lymphocytic infiltration and an absence of significant necrosis (arrowhead). **(B)** Diabetic control (40x) exhibiting severe injury, including marked lymphocytic infiltration, architectural distortion, and multifocal hepatocyte necrosis (arrowhead). **(C)** Normal control (10x) demonstrating intact physiological architecture without pathology. **(D**–**F)** Representative H&E-stained kidney sections (40x). **(D)** 600 mg/kg treatment showing perivascular infiltrates and cytoplasmic vacuolization of tubular epithelial cells (arrowhead) with preserved glomeruli. **(E)** Diabetic control with prominent perivascular lymphocytic infiltrates and cytoplasmic vacuolization (arrowhead). **(F)** Normal control (10x) showing intact glomeruli and tubules with a mild, non-specific inflammatory infiltrate. **(G**–**I)** Representative H&E-stained cardiac sections. **(G)** 600 mg/kg treatment showing preserved myocardial architecture with no significant drug-related pathology (arrowhead). **(H)** Diabetic control (40x) revealing severe perivascular pathology, including foam cell accumulation (arrowhead), luminal thrombus, and hemorrhagic foci. **(I)** Normal control demonstrating preserved architecture and normal cardiomyocytes (arrowhead). Scale bars are as indicated in each panel.

The 600 mg/kg dose group exhibited a significantly attenuated pathological profile (Fig. 4-A). The hepatic architecture was well-preserved, with intact central veins and portal triads. Pathological findings were limited to mild perivascular lymphocytic infiltration, with a notable absence of the necrosis, sinusoidal distortion, and cellular stress markers observed at lower doses. This suggests a potential non-linear or hormetic response that requires further investigation.

The diabetic control group (Fig. 4-B) exhibited severe hepatitis, providing a baseline of disease-associated injury. Features included marked perivascular lymphocytic infiltration, lymphocyte aggregates, severe architectural distortion, and widespread multifocal necrosis, highlighting the significant underlying pathology of the disease model.

In contrast, the positive control group showed a largely preserved hepatic architecture with only minimal focal necrosis and scattered debris. The marked reduction in inflammatory infiltrates and necrosis, compared to the diabetic control, validated the efficacy of the standard treatment and the experimental system.

Finally, the normal control group (Fig. 4-C) established the standard for healthy histology, demonstrating perfectly preserved architecture and a complete absence of all pathological features, confirming the absence of non-specific procedural injury.

3. Kidney Morphology

Histopathological assessment of kidney tissues revealed a distinct pattern of injury. At 200 mg/kg, sections exhibited mixed acute and chronic changes, including perivascular inflammatory infiltrates, cytoplasmic vacuolization of tubular epithelial cells (indicating cellular stress), and vascular hyalinization. The overall glomerular and tubular architecture remained preserved indicating a mixed acute inflammatory and chronic vascular toxicological response distinct from hepatic injury.

The 400 mg/kg dose resulted in a clear progression of pathology. The inflammatory response intensified, with multiple prominent foci of perivascular lymphocytic inflammation, while tubular epithelial vacuolization persisted. The preservation of renal architecture indicated that the damage remained inflammatory and degenerative rather than destructive at this dose. In the 600 mg/kg dose, the acute inflammatory pattern persisted, characterized by perivascular infiltrates and consistent tubular epithelial vacuolization. The preservation of glomerular and tubular architecture indicated a lack of overt necrosis or fibrosis (Fig. 4-D). The ubiquitous presence of vacuolization across all doses suggests it is a primary, dose-independent cellular response to the compound, reinforcing the conclusion that it induces a specific acute tubulointerstitial nephritis and vascular inflammation.

Kidney sections from the diabetic control group established the baseline disease pathology, showing pronounced perivascular lymphocytic infiltrates and tubular vacuolization, with preserved architecture characteristic of diabetic nephropathy and interstitial inflammation (Fig. 4-E).

Notably, the positive control (glibenclamide) group exhibited renal pathology nearly identical to the diabetic control, characterized by prominent perivascular inflammation and tubular vacuolization. This lack of renal protection by the standard drug underscores the specific efficacy of ZAE in mitigating diabetic nephropathy in this model.

The normal control group established the standard for healthy renal architecture, with intact glomeruli and renal tubules (Fig. 4-F). A mild, background lymphocytic infiltrate was present but is considered a normal finding in rodent histology, providing a baseline for distinguishing normal variation from significant pathology.

4) Heart Morphology

Histological evaluation revealed a stark contrast in cardiac tissue compared to the liver and kidney, indicating a favorable cardiac safety profile for the experimental compound. Myocardial architecture was well-preserved in the positive control and all treatment groups 200, 400, and 600 mg/kg, with particularly notable preservation observed at the 600 mg/kg dose (Fig. 4-G). Cardiomyocytes appeared normal, with a complete absence of pathological findings such as myofiber degeneration, necrosis, or inflammatory infiltrates at all administered doses. The normal control group (Fig. 4-I) displayed the expected healthy architecture, providing a baseline.

In contrast, cardiac tissue from the diabetic control group (Fig. 4-H) exhibited severe vascular pathology, a known complication of diabetes. Key features included prominent foam cell accumulation, indicative of advanced lipid deposition and atherogenesis, accompanied by luminal thrombus formation and hemorrhagic foci. These changes are consistent with significant diabetic vascular compromise. Crucially, the underlying cardiomyocytes maintained normal architecture, indicating that the primary diabetic insult is vascular rather than a direct toxic effect on the myocardium.

This finding underscores the severity of the diabetic model and demonstrates that the test compound does not induce direct cardiotoxicity or exacerbate the underlying diabetic vascular disease.

### 3.5 Organ weight analysis

Absolute organ weights for the pancreas, liver, kidneys, and heart are presented in Table 4.

**Table 4:** Organ weights across experimental groups in STZ-Induced diabetic mice.

Group	Pancreas (mg)	Liver (mg)	Kidney (mg)	Heart (mg)
Normal Ctrl	282.8 ± 48.6	1210.2 ± 187.9	311.8 ± 9.0	115.7 ± 12.3
(N = 6)	(223–362)	(1000–1421)	(300–324)	(100–130)
Diabetic Ctrl	145.7 ± 14.4∗∗∗	2452.5 ± 250.5∗∗∗	416.5 ± 33.4∗∗∗	197.7 ± 12.3∗∗∗
(N = 6)	(125–162)	(2100–2698)	(386–480)	(180–212)
Positive Ctrl	189.7 ± 25.1∗∗	2035.3 ± 112.9∗∗∗	316.3 ± 48.2	155.2 ± 4.6∗∗∗
(N = 6)	(150–214)	(1894–2223)	(257–385)	(149–162)
Low Dose	159.8 ± 9.4∗∗∗	2198.3 ± 421.0∗∗∗	373.3 ± 13.1∗	120.2 ± 12.4
(N = 6)	(148–172)	(1500–2600)	(359–394)	(102–135)
Middle Dose	200.7 ± 18.5∗	2192.7 ± 314.8∗∗∗	333.8 ± 28.5	104.8 ± 7.7
(N = 6)	(180–234)	(1899–2700)	(300–379)	(100–120)
High Dose	267.8 ± 42.4∗∗	1677.5 ± 213.7∗	308.8 ± 40.4	113.8 ± 9.5
(N = 6)	(200–310)	(1400–1987)	(279–389)	(98–125)

Data presented as Mean ± SD (range)**.** Sample size: *N* = 6 mice/group. Statistical significance was determined by one-way ANOVA with Tukey's post-hoc test. ∗*P* < 0.05, ∗∗*P* < 0.01, ∗∗*P < 0.001* vs. Normal Control.

**Pancreas:** The absolute pancreas weight of the Diabetic control group (145.7 ± 14.4 mg) was significantly lower (*P* < 0.001) than that of the Normal control group (282.8 ± 48.6 mg). The pancreas weight of the ZAE 600 mg/kg group (267.8 ± 42.4 mg) was significantly higher than that of the Diabetic control (*P* < 0.01) and was not significantly different from the Normal control.

**Liver:** The Diabetic control group exhibited a significant increase in liver weight (2452.5 ± 250.5 mg, *P* < 0.001) compared to the Normal control (1210.2 ± 187.9 mg). This increase was significantly reduced in the Positive Control (Glibenclamide, 5 mg/kg) group (2035.3 ± 112.9 mg, *P* < 0.001 vs. Diabetic control) and the ZAE 600 mg/kg group (1677.5 ± 213.7 mg, *P* < 0.05 vs. Diabetic control).

**Kidneys:** Kidney weight was significantly higher in the Diabetic control group (416.5 ± 33.4 mg, *P* < 0.001) compared to the Normal control (311.8 ± 9.0 mg). Kidney weight in the Positive Control group (316.3 ± 48.2 mg) was not significantly different from the Normal control. The ZAE 200 mg/kg group kidney weight (373.3 ± 13.1 mg) remained significantly elevated compared to the Normal control (*P* < 0.05), while the ZAE 600 mg/kg group kidney weight (308.8 ± 40.4 mg) was not significantly different from the Normal control.

**Heart:** The Diabetic control group had a significantly higher heart weight (197.7 ± 12.3 mg, *P* < 0.001) than the Normal control (115.7 ± 12.3 mg). The heart weights of the ZAE 200, 400, and 600 mg/kg groups (120.2 ± 12.4 mg, 104.8 ± 7.7 mg, and 113.8 ± 9.5 mg, respectively) were not significantly different from the Normal control. The heart weight of the Positive Control group (155.2 ± 4.6 mg) remained significantly elevated compared to the Normal control (*P* < 0.001).

## 4 Discussion

This explorative study reveals that the 600 mg/kg treatment with *Ziziphus abyssinica* methanolic extract (ZAE) provides a comprehensive therapeutic profile, extends beyond glycemic control to encompass significant, dose-dependent multiorgan protection a comprehensive evaluation not commonly reported in initial screenings of medicinal plants. It is important to note the context of our experimental model. The multi-low-dose STZ protocol primarily induces a type 1-like diabetic state characterized by β-cell destruction. The choice of glibenclamide, a sulfonylurea used for type 2 diabetes, as a positive control was to benchmark a standard insulin secretagogue. Its limited efficacy in this model underscores the severity of β-cell loss. In contrast, the significant restoration of islet morphology and β-cell density by high-dose ZAE suggests its mechanisms extend beyond stimulating insulin secretion to include β-cell cytoprotection and/or regeneration. Our findings corroborate its traditional use in African ethnomedicine [46], and expand upon prior work on *Ziziphus species* (e.g., *Z. mucronata*), which primarily reported hypoglycemic and antioxidant properties [11]. Uniquely, this study establishes a clear dose-response relationship for both glycemic control and organ-specific protection within a standardized STZ model [13], highlighting ZAE's capacity for pancreatic β-cell preservation and concurrent mitigation of hepatorenal and cardiac damage.

The dose-dependent reduction in blood glucose levels with ZAE aligns with studies [47] on other flavonoid-rich botanicals (e.g., *Berberis aristata*) that modulate AMPK and hepatic gluconeogenesis [17]. However, while glibenclamide acts primarily as an insulin secretagogue, ZAE's effects appear multimodal, including the significant restoration of pancreatic β-cell density, an effect not typically associated with sulfonylureas [6]. This suggests ZAE's multimodal action both insulin-sensitizing and β-cell protective mirrors mechanisms observed in *Gymnema sylvestre* [7], but with greater histopathological recovery.

The partial restoration of islet morphology in ZAE-treated mice parallels findings with *Ocimum sanctum* extracts [15]. though ZAE uniquely reduced insulitis severity**.** This immunomodulatory potential distinguishes it from pure antioxidants [48] like quercetin, which show weaker anti-inflammatory effects in STZ models [13]. A notable finding was the non-linear, dose-dependent effect of ZAE on the liver. Although inflammatory markers and architectural distortion were observed at 200 and 400 mg/kg, the 600 mg/kg resulted in well-preserved hepatic architecture and a significant attenuation of pathology. This suggests a potential threshold or hormetic effect where the extract's restorative antioxidant and anti-inflammatory properties overcome any initial stress response, resulting in net hepatoprotection at the therapeutic dose. Liver preservation in high-dose groups mirrors berberine's lipid-regulating effects [17]. but ZAE's additional attenuation of hepatomegale (Table 4) suggests broader metabolic benefits [49].

Furthermore, ZAE's efficacy extended to organs where glibenclamide showed limited or no protective effect in our model. Most notably, ZAE attenuated diabetic nephropathy, as evidenced by reduced tubular vacuolization, while glibenclamide treatment failed to show significant renal protection. Additionally, ZAE treatment normalized cardiac weight and prevented foam cell accumulation, effects not observed with glibenclamide.

ZAE's reduction of tubular vacuolization and glomerular damage aligns with nephroprotective effects of *Momordica charantia* [50]. yet its concurrent normalization of cardiac weight (Table 4) and lipid profiles is rare among plant extracts [51]. While metformin improves lipid metabolism [6], ZAE's foam cell reduction indicates direct anti-atherogenic activity a feature shared only by a few botanicals like *Allium sativum* [5].

Compared to clinical trials on established antidiabetic herbs (e.g., *Cinnamomum cassia* [20], this study is limited to murine models**.** Future work should: Compare ZAE's bioactive compounds (e.g., spinosin) to isolated flavonoids like rutin for target specificity [7], Evaluate long-term safety, as hepatorenal protection in mice may not fully translate to humans [15]. Test combinatorial therapies with synthetics (e.g., low-dose ZAE + metformin) to optimize efficacy [6], Future studies should isolate and characterize specific bioactive compounds in *Ziziphus abyssinica* to determine their individual contributions to antidiabetic effects. Comparative studies with isolated compounds like rutin or quercetin could elucidate structure-activity relationships and synergistic interactions [11].

While ZAE demonstrated hepatorenal protection in mice, long-term toxicity studies are essential to assess its safety for human use. Chronic toxicity evaluations in higher mammalian models, including histopathological and biochemical monitoring, would bridge the translational gap [20,43].

Investigating ZAE in combination with synthetic drugs (glibenclamide) could optimize glycemic control while minimizing adverse effects. Such approaches may leverage ZAE's β-cell regenerative properties alongside conventional insulin sensitizers [6,7].

Clinical trials are needed to validate ZAE's efficacy in human populations, particularly in regions where *Ziziphus species* are traditionally used. Ethnopharmacological surveys could identify optimal dosing regimens and patient-specific responses [2,10].

Further mechanistic studies should explore ZAE's effects on oxidative stress pathways (e.g., Nrf2 activation) and immunomodulation (e.g., TNF-α suppression) to clarify its multimodal action. Proteomic or transcriptomic analyses could reveal novel targets [5,16].

## 5 Conclusion

This study provides robust experimental evidence that 600 mg/kg *Ziziphus abyssinica* methanolic extract (ZAE) acts as a potent antidiabetic agent with concurrent multi-organ protective effects. Despite mixed effects at 200 mg/kg and 400 mg/kg, the 600 mg/kg treatment consistently ameliorated hyperglycemia, restored pancreatic β-cell mass, and mitigated diabetes-associated damage in the liver, kidney, and heart.

Notably, ZAE coincided with glibenclamide in pancreatic islet regeneration and exhibited multimodal organ protection, including. Normalization of hepatomegaly and reduction in oxidative stress markers (ALT, AST, ALP). Attenuation of diabetic nephropathy, evidenced by preserved glomerular architecture and reduced tubular vacuolization. Prevention of hypertrophy and foam cell accumulation, suggesting cardioprotective potential. These findings validate ethnomedicinal claims while providing a scientific basis for further research into clinical translation via human trials to assess safety and efficacy. Mechanistic studies to isolate bioactive compounds (e.g., spinosin) and elucidate pathways (e.g., AMPK modulation, Nrf2 activation).

*Ziziphus abyssinica* represents a promising candidate for plant-based diabetes management**,** offering a holistic alternative to current treatments by addressing both metabolic dysregulation and diabetic complications. Future work should prioritize standardization, toxicity profiling, and ethnopharmacological validation to bridge traditional knowledge with modern therapeutics.

## Declaration of generative AI in scientific writing

During the preparation of this work, the authors used DeepSeek and Grammarly solely for the purpose of post-editing and polishing the author-written text. This assistance was limited to improving language, checking grammar, spelling, and enhancing sentence clarity and readability. After using these tools, the authors reviewed and edited all content as needed and take full responsibility for the scientific content of the publication. All scientific ideation, data generation, analysis, interpretation, and the initial drafting of the manuscript were performed by the authors.

## Sources of funding

This research was supported by internal funding from the Armauer Hansen Research Institute (AHRI) and Addis Ababa University (AAU), Ethiopia under grant number AAU-AHRI-2024/25. The funding organization had no role in the design of the study, data collection, analysis, or interpretation of the results.

## Conflict of interest

The authors declare that they have no known competing financial interests or personal relationships that could have appeared to influence the work reported in this paper.

## Figures and Tables

**Fig. S1:**
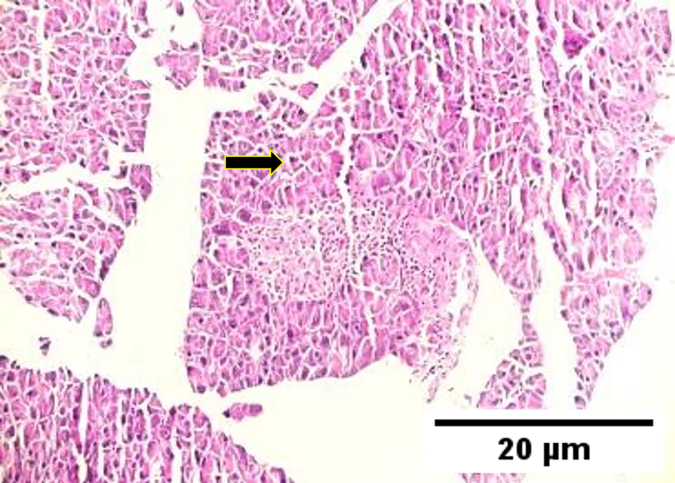
**Pancreatic histology in an STZ-induced diabetic mouse treated with 200 mg/kg ZAE.** Representative H&E-stained section (40× magnification) of the pancreas. The **arrowhead** points to a representative pancreatic islet, which is delineated from the surrounding exocrine (Ex) tissue. Scale bar: 20 μm.

**Fig. S2:**
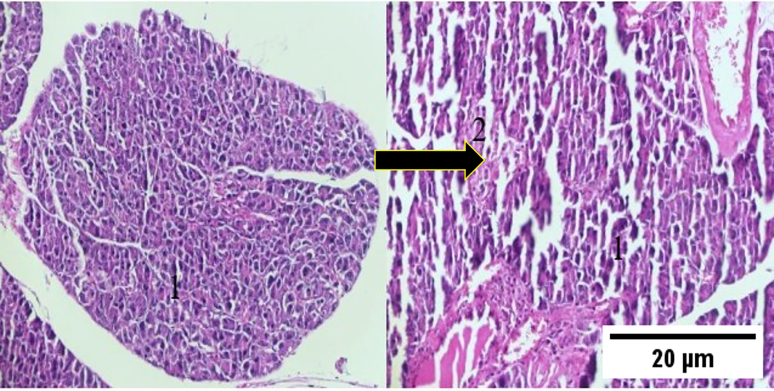
**Pancreatic histology of STZ-induced diabetic mice treated with 400 mg/kg ZAE.** Representative H&E-stained pancreatic section (40× magnification) from **mice** treated with STZ followed by **400 mg/kg ZAE**. The image shows the exocrine pancreas, and a representative islet of Langerhans. Pathological features include islet shrinkage, reduced cellularity within the islet **(arrowhead)**, and the presence of lymphocytic infiltration (insulitis). Scale bar, 20 μm.

**Fig. S3:**
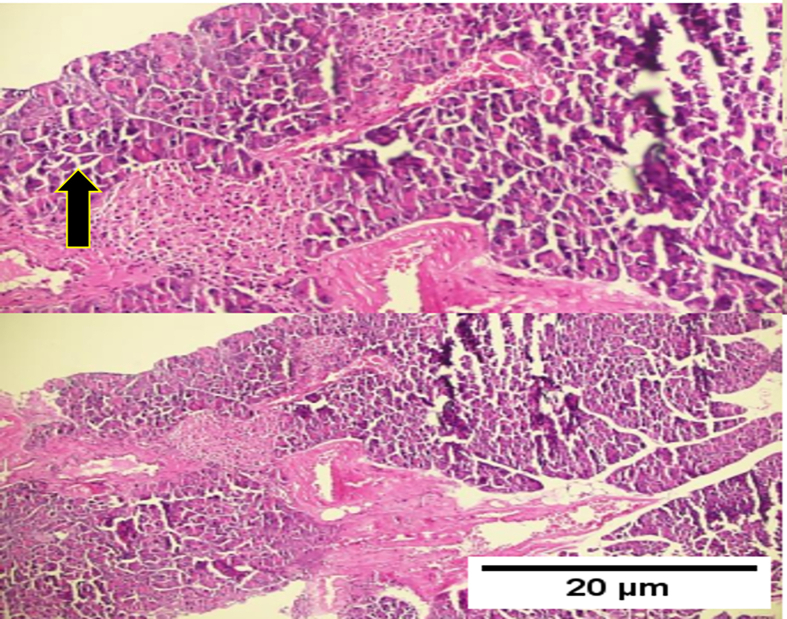
**Pancreatic histology of an STZ-induced diabetic mice treated with glibenclamide.** Representative H&E-stained pancreatic section from an STZ-induced diabetic **mice** treated with **5 mg/kg glibenclamide** (40× magnification). The image shows exocrine pancreatic acini and a representative islet of Langerhans**(arrowhead)**. The islet architecture and cellularity are more preserved relative to the untreated diabetic control group (Fig. S2). Scale bar, 20 μm.

**Fig. S4:**
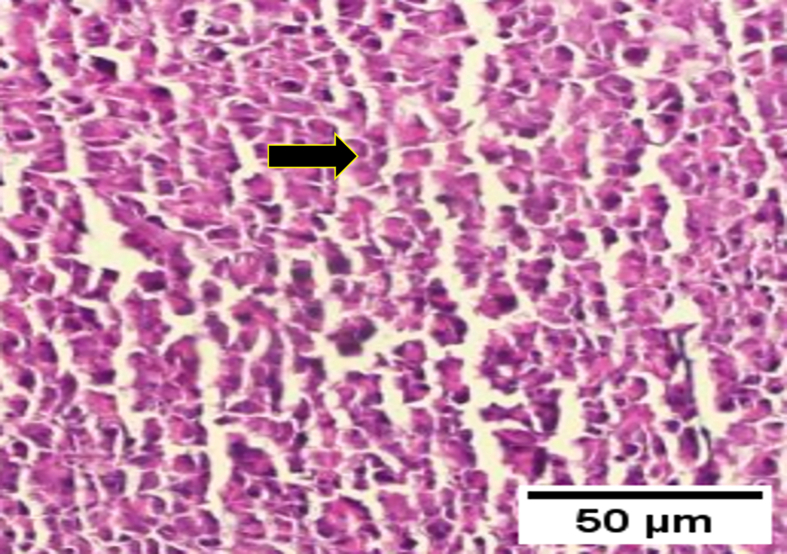
**Pancreatic histology of a non-diabetic healthy control mouse.** Representative H&E-stained pancreatic section from non-diabetic healthy control **mice** (10× magnification). The image shows the characteristic architecture of the exocrine pancreatic acini and a large, well-defined islet of Langerhans **(arrowhead)** with high cellular density. Scale bar, Scale bar, 50 μm.

**Fig. S5:**
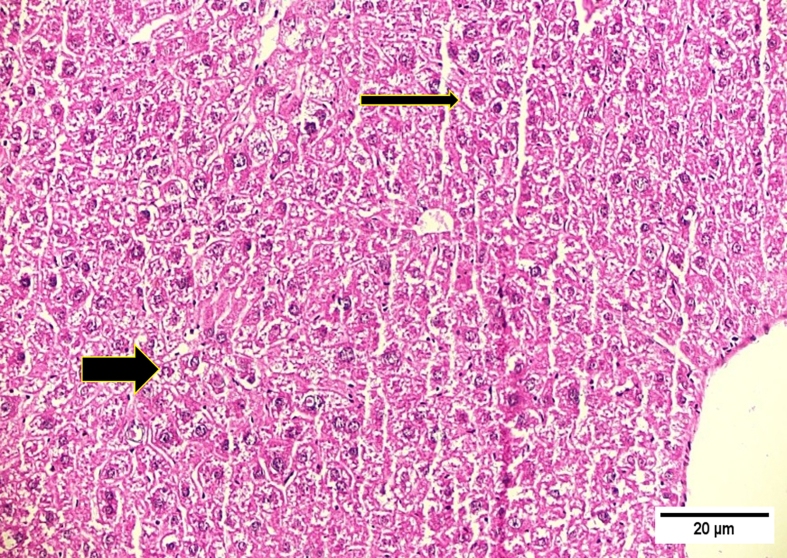
**Histopathological features of liver injury in a low-dose (200 mg/kg) treatment model.** Representative photomicrograph of a hematoxylin and eosin (H&E)-stained liver section (40× magnification) from a treated mouse. Key findings include: (1) lymphocytic infiltration in the portal and periportal regions; (2) dilated sinusoids containing microvesicular fatty changes (thin arrows); (3) distorted hepatic architecture with foci of necrotic hepatocytes (thick arrows) admixed with lymphocytes; and (4) the presence of sinusoidal cell debris, hyperchromatic nuclei, and pyknotic nuclei (indicative of cellular stress). Scale bar: 20 μm.

**Fig. S6:**
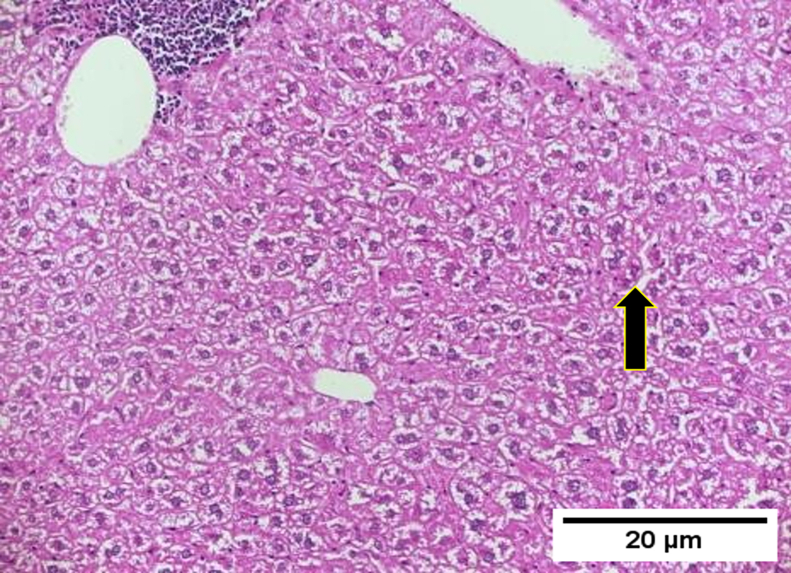
**Progression of histopathological liver injury in a middle-dose (400 mg/kg) treatment model.** Representative photomicrograph of a hematoxylin and eosin (H&E)-stained liver section (40× magnification) from a treated mouse. Histological analysis demonstrates an exacerbation of injury, featuring: (1) prominent perivascular lymphocytic infiltration and dilated sinusoids with microvesicular steatosis; (2) significantly distorted hepatic architecture with extensive foci of necrotic hepatocytes admixed with lymphocytes and sinusoidal cell debris; and (3) clear markers of cellular stress, including hyperchromatic nuclei and pyknosis. Notably, this dose also induces perivenular inflammation surrounding the dilated central vein **(arrowhead)**. Scale bar: 20 μm.

**Fig. S7:**
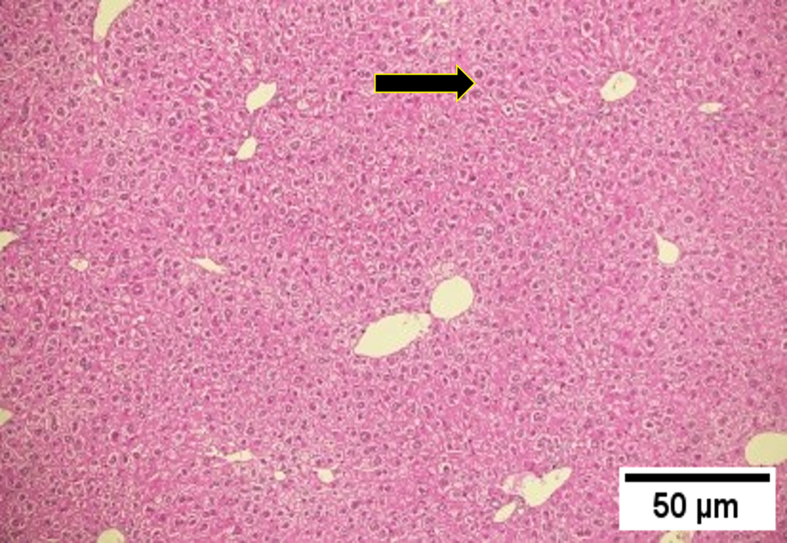
**Histopathological assessment of liver tissue in a positive control treatment model.** Representative photomicrograph of a hematoxylin and eosin (H&E)-stained liver section (10× magnification) from a positive control mouse. The lobular architecture is well-preserved with intact central veins and portal triads. Focal changes are present, including subtle foci of hepatocyte necrosis and scattered cellular debris within the sinusoids **(arrowhead)**. Scale bar: 50 μm.

**Fig. S8:**
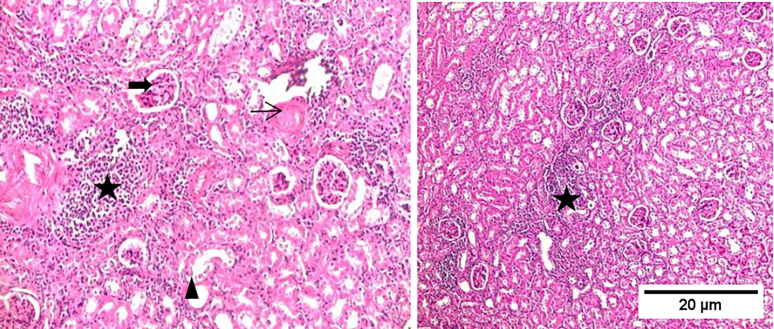
**Histopathological features of kidney injury in a low-dose (200 mg/kg) treatment model.** Representative photomicrograph of a hematoxylin and eosin (H&E)-stained kidney section (40× magnification) from a treated mouse. The section demonstrates: (1) perivascular inflammatory infiltrates (Stars); (2) vascular hyalinization (indicative of chronic vascular injury); and (3) cytoplasmic vacuolization of tubular epithelial cells (arrowhead). The overall glomerular and tubular architecture remains preserved. These combined features indicate concurrent acute inflammatory and chronic vascular injury pathways. Scale bar: 20 μm.

**Fig. S9:**
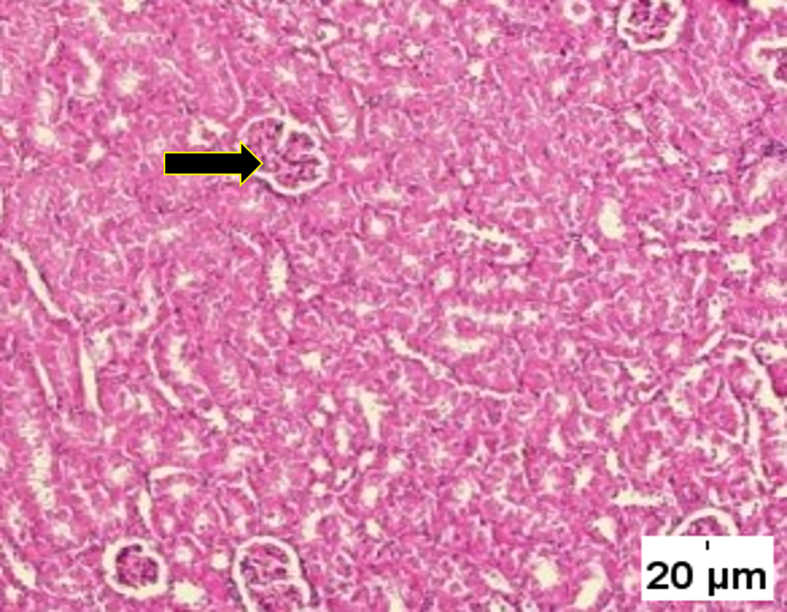
**Progression of kidney injury in a middle-dose (400 mg/kg) treatment model.** Representative photomicrograph of a hematoxylin and eosin (H&E)-stained kidney section (40× magnification) from a treated mouse. The section shows an intensification of damage, featuring: (1) multiple prominent foci of perivascular lymphocytic inflammation; and (2) cytoplasmic vacuolization in the tubular epithelial cells **(arrowhead)**. Despite these injuries, the glomeruli and renal tubules remain preserved at this dose. Scale bar: 20 μm.

**Fig. S10:**
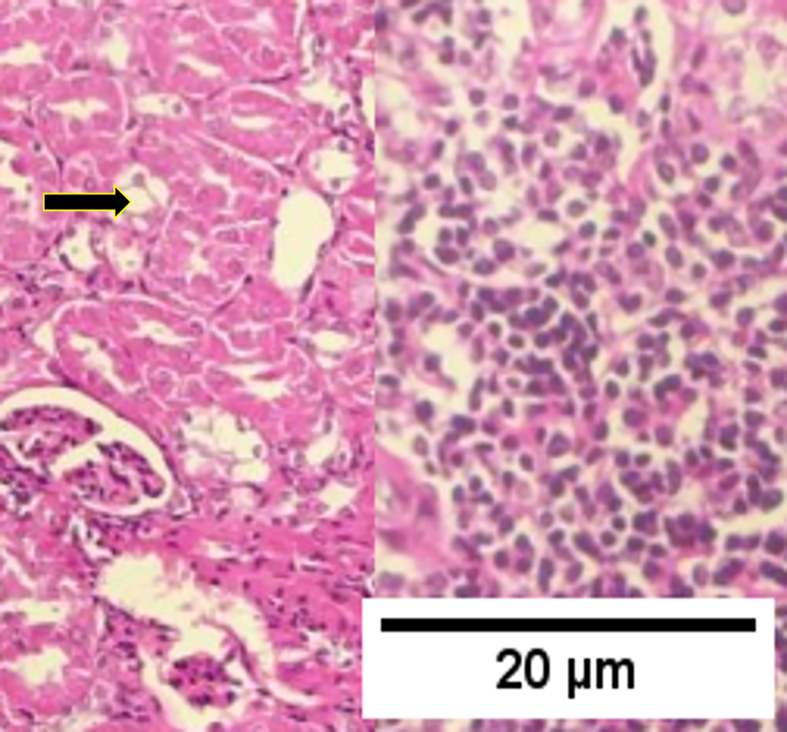
**Kidney histopathology in a positive control treatment model.** Representative photomicrograph of a hematoxylin and eosin (H&E)-stained kidney section (40× magnification) from a positive control mouse. The kidney displays: (1) multiple prominent foci of perivascular lymphocytic inflammation; and (2) cytoplasmic vacuolization in tubular epithelial cells**(arrowhead)**. The architecture of the glomeruli and renal tubules remains preserved. Scale bar: 20 μm.

**Fig. S11–S13:**
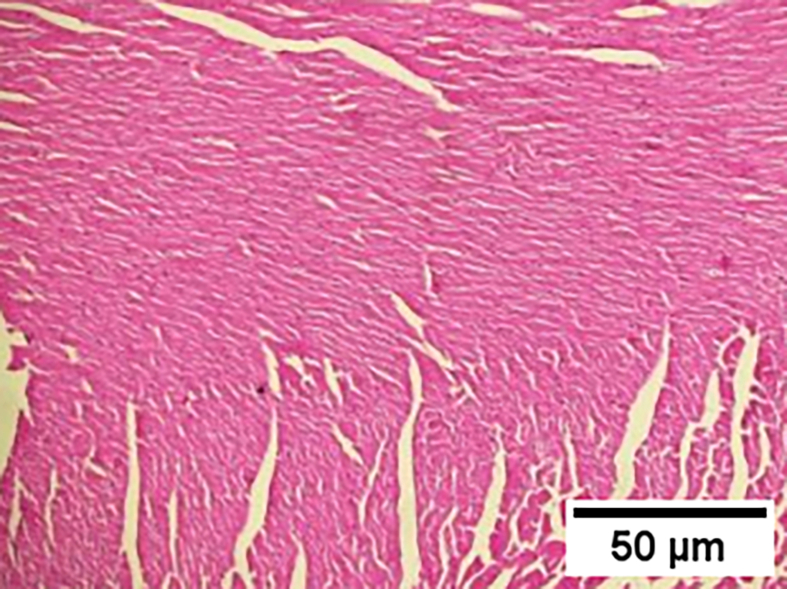
**Histopathological assessment of cardiac tissue across all experimental groups.**Representative photomicrographs of hematoxylin and eosin (H&E)-stained cardiac sections. Fig. S11 (200 mg/kg), S12 (400 mg/kg), S13 (positive control), and all demonstrate preserved myocardial architecture with nearly normal cardiomyocytes, showing no significant drug-related pathological changes. Scale bars: 20 μm (for 40x images) and 50 μm (for 10x images).

**figs12:**
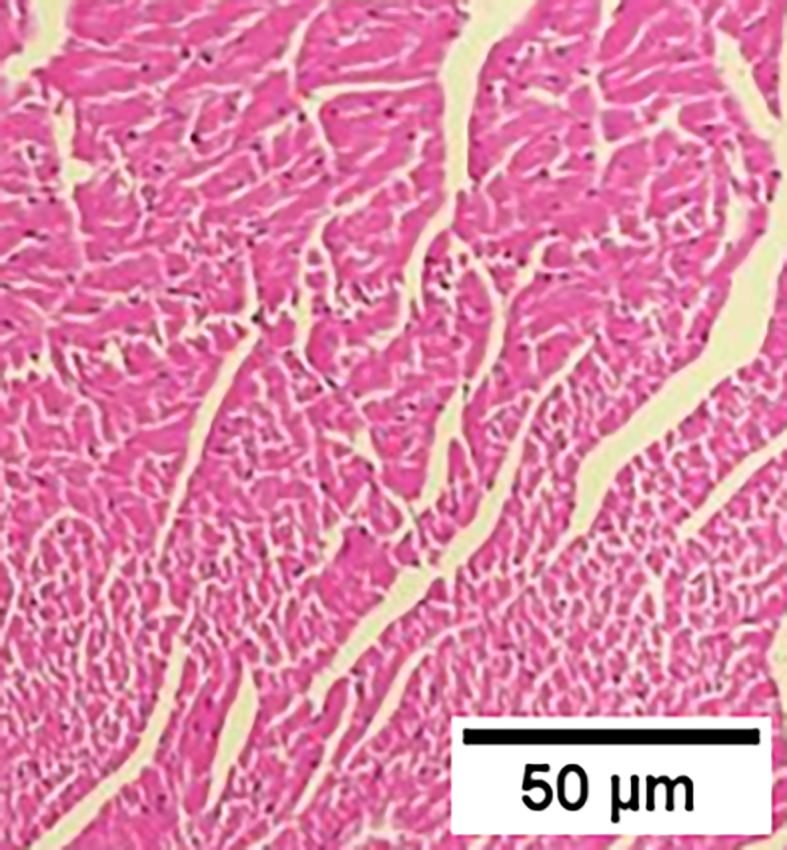


**figs13:**